# A Moonlighting Function of *Plasmodium falciparum* Histone 3, Mono-Methylated at Lysine 9?

**DOI:** 10.1371/journal.pone.0010252

**Published:** 2010-04-19

**Authors:** Yen-Hoon Luah, Balbir Kaur Chaal, Eugenia Ziying Ong, Zbynek Bozdech

**Affiliations:** School of Biological Sciences, Nanyang Technological University, Singapore, Singapore; Institute of Infectious Disease and Molecular Medicine, South Africa

## Abstract

**Background:**

In the human malaria parasites *Plasmodium falciparum*, histone modifications have been implicated in the transcriptional regulation. The acetylation and methylation status of the histones have been linked with transcriptional regulation of the parasite surface virulence factors as well as other genes with stage specific expression. In *P. falciparum* as well as other eukaryotes, different histone modifications were found to be compartmentalized to distinct regions in the nuclei. This compartmentalization is believed to be one of the main prerequisites for their function in epigenetic regulation of gene expression.

**Methodology/Principal Findings:**

Here we investigate intracellular distributions of five previously uncharacterized histone modifications including histone 4 acetylation on lysine residue 5 (H4K5Ac), H4K8Ac, H3K9Ac, H4Ac4 and H3K9Me1 during the asexual developmental stages. With the exception of H3K9Me1, the modified histones were localized to the nuclear periphery. This provides a strong indication that the *P. falciparum* nuclear periphery is one of the most active regions in epigenetic regulation of gene expression. Interestingly, H3K9Me1 is not associated with the nuclei but instead resides in the parasitophorous vacuole (PV), the double membrane compartments surrounding the parasite cell within the host erythrocyte. In this compartment, H3K9Me1 partially co-localizes with Etramp proteins. The localization of H3K9Me1 in the PV is conserved in the other species including *P. yoelii* and *P. vivax*.

**Conclusions:**

Similar to other eukaryotes, the periphery of the *P. falciparum* nuclei is likely one of the most active areas in epigenetic regulation of gene expression involving multiple histone modifications. On the other hand, H3K9Me1 evolved a new function that is linked with the PV. This functional role appears to be evolutionarily conserved in *Plasmodium* species.

## Introduction

Similar to other eukaryotic organisms, in the human malaria parasite *Plasmodium falciparum*, histone modifications have been implicated in chromatin remodeling and transcriptional regulation [1], [2], [3], [4], [5], [6]. All 7 genes that encode the core *P. falciparum* nucleosome subunits are highly homologous to their eukaryotic counterparts including human [7]. These include one homologue of histone 4 (PfH4) and two homologues of histone 3 (PfH3 and PfH3.3), histone 2A (PfH2A and PfH2A.Z) and histone 2B (PfH2B and PfH2Bv). Initial studies using tandem mass spectrometry of nucleosomes extracted from the intraerythrocytic developmental stages of the *P. falciparum* parasites uncovered acetylations and methylation of lysine and arginine residues at the well conserved N-termini of all seven histones [8]. These findings indicated an important role of histone modifications in gene expression that regulates the progression of the *Plasmodium* life cycle, as well as the growth, virulence and interactions with its host.

Histone modifications are likely involved in global regulation of *P. falciparum* gene expression that is associated with the progression of the life cycle. In a recent study, Salcedo-Amarya et al (2009) found two histone modifications H3K4Me3 and H3K9Ac, enriched at the 5′ regions of transcriptionally active genes in the later developmental stage (schizonts) of *P. falciparum* [6]. This study followed a previous report in which a histone acetyltransferase was found to be responsible for acetylation of H3K9 at putative transcriptional initiation sites and implicated in transcriptional regulation of *Plasmodium* genes [2].

Extensive studies of epigenetic silencing of subtelomericaly located variable surface antigen (VSA) families, namely the *var* gene family, have also been carried out. Here it was shown that epigenetic markers represented by specific histone modifications including both acetylation and methylation, were associated with the mutually exclusive expression of individual *var* genes [3], [4], [5], [9]. Lopez-Rubio (2007) demonstrated that the *var* gene silencing is mediated by tri-methylation of histone 3 at lysine residue 9 (H3K9Me3) [10] which concentrates in distinct compartments at the nuclear periphery [11]. In addition, at least additional three distinct pattern of nuclear distribution were found for other types of histone modifications [11]. A continuous “horseshoe-like” pattern around the nuclear periphery was observed for H3K4Me3, and H4K20Me3, typically markers of gene activation and repression, respectively [12]. In contrast, H3K4Me2 another activation marker showed a strong punctuate distribution spread throughout the nuclei. The most striking pattern was observed for H3K79Me3 that was characterized by 3-5 distinct dots clustered at a one end on the nuclei [11]. Taken together, these data suggested that the localization may be a major prerequisite for specific biological roles of the histone isoforms in gene expression, marking distinct regions of chromatin distribution in the nuclei. Besides the role of the H3K9Me3 in the silencing of the subtelomeric gene families that has been linked with the two perinuclear foci, close to nothing is known about the biological significance of the different subnuclear compartments.

Here we aim to characterize intracellular distributions of several previously uncharacterized histone modifications including four types of acetylations H4K5Ac, H4K8Ac, H3K9Ac and H4K5,8,12,16 tetraAc (H4Ac4) and one methylation H3K9Me1. The protein distribution pattern of the studied histone acetylations resembles the horseshoe-like pattern observed for H3K4Me3 [11] suggesting their overlapping roles in gene expression. Intriguingly, H3K9Me1 is not localized to the nuclei but it is mainly transported to the parasite periphery where it partly colocalizes with proteins of the parasitophorous vacuole (PV). The PV localization is evolutionarily conserved in other *Plasmodium* species which strongly indicates that histone 3 may have evolved an additional function that is associated directly with parasite invasion and/or interaction with the host.

## Results

### Localization of histone modifications in *P. falciparum*

**Figure 1:**
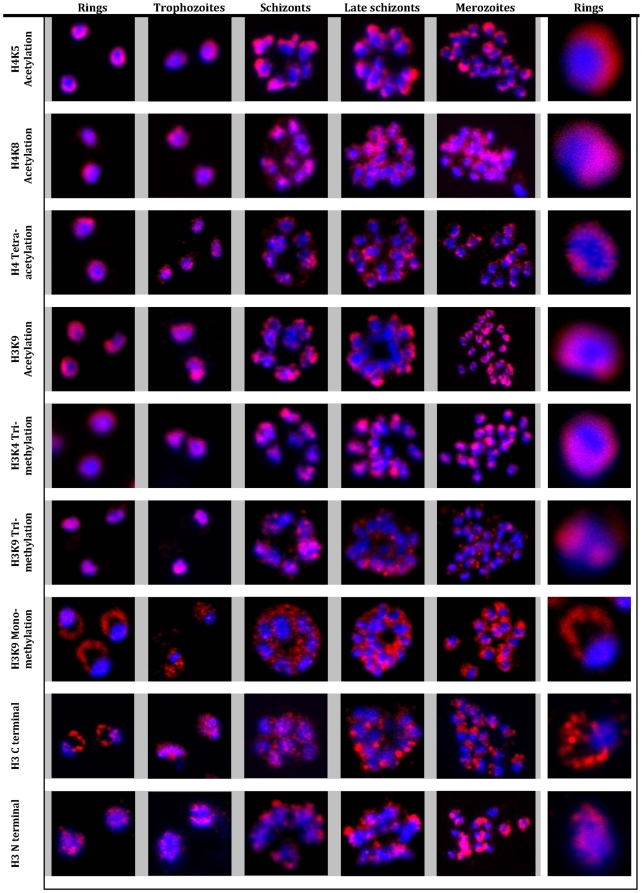
Immunofluorescence analysis of histone modifications in *P. falciparum*. Localization of histone modifications were analyzed with the ring, trophozoite, schizont, late schizont and merozoite stages of the IDC. IFAs were carried out with antibodies against specific histone 3 and 4 lysine residue acetylations: H3K9Ac, H4K5Ac, H4K8Ac, and H4Ac4, as well as methylations: H3K4Me3, H3K9Me3 and H3K9Me1, and unmodified histone H3. Nuclear DNA was stained with DAPI (blue). All modifications, with the exception of H3K9Me1 and H3 (antibody raised against H3 C terminal), showed specific and distinct localization in the nucleus in all the stages. In contrast, H3K9Me1 was localized mainly outside the nucleus with very low levels detected inside the nucleus.

For the intracellular localizations, we carried out indirect immunofluorescence microscopy (IF) with monoclonal antibodies raised against the specific epitopes corresponding to the studied histone modifications. To gain insights into the role of histone modifications in the progression of the *Plasmodium* life cycle, the IFs were conducted with four distinct developmental stages of the 48 hour intraerythrocytic developmental cycle (IDC), ring, trophozoite, schizont and merozoite (Figure 1). In the ring stage, H4K5Ac, H4K8Ac, H4Ac4 and H3K9Ac exhibited a perinuclear, horseshoe-like distribution pattern, similar to the one previously reported for H3K4Me3 [11]. For H4Ac4 and H3K9Ac, the perinuclear distribution was preserved throughout the entire IDC. For H4K5Ac and H3K4Me3, there was an increased apparent polarization of the IF signal at a distinct region at the nuclear periphery with the progression of the IDC. In contrast, the IF pattern of H4K8Ac became progressively irregular with the progression of the IDC, appearing as fibrous-like structures accompanied by a diffused pattern distributed throughout the nuclei (Figure 1). As a control, we also carried out localizations of two previously characterized histone modifications, H3K4Me3 and H3K9Me3. Both the horseshoe-like pattern of H3K4Me3 and the two-foci distribution of H3K9Me3 previously reported in the trophozoite stage [11], were preserved throughout the IDC (Figure 1).

The most striking pattern of intracellular localization was observed for H3K9Me1. Although a small portion of the IF signal could be detected in the nuclei (data not shown), the vast majority of this histone modification localized outside the nuclei (Figure 1). The crescent like pattern in the ring stage suggests that at this stage, H3K9Me1 is transported to the periphery of the parasite cells and is potentially exported beyond its plasma membrane. In the trophozoite and schizont stages, the H3K9Me1 pattern became more punctuate but remained distant from the nuclear compartment. In the released merozoites, H3K9Me1 appeared to reside outside of the nuclei, polarized towards one side of the merozoite cytoplasm (Figure 1). This pattern is exclusive to mono-methylation of lysine residue 9 since acetylation at this residue (H3K9Ac) results in the horse-shoe like distribution (Figure 1). The crescent like IFA pattern was also observed in an experiment carried out with the core H3 antibody that recognizes an epitope in the C-terminus on the histone protein (Figure 1, H3 C terminal). On the other hand, IFA signal with H3-N-terminal antibody was found strictly in the nucleus. This antibody recognizes the identical epitope to the H3K9Me1 and H3K9Ac antibody that is devoid of all modifications. These results further confirm that the extracellular H3 protein is mono-metylated at the lysine residue 9 (Figure1).

### H3K9Me1localized to the parasitophorous vacuole

**Figure 2:**
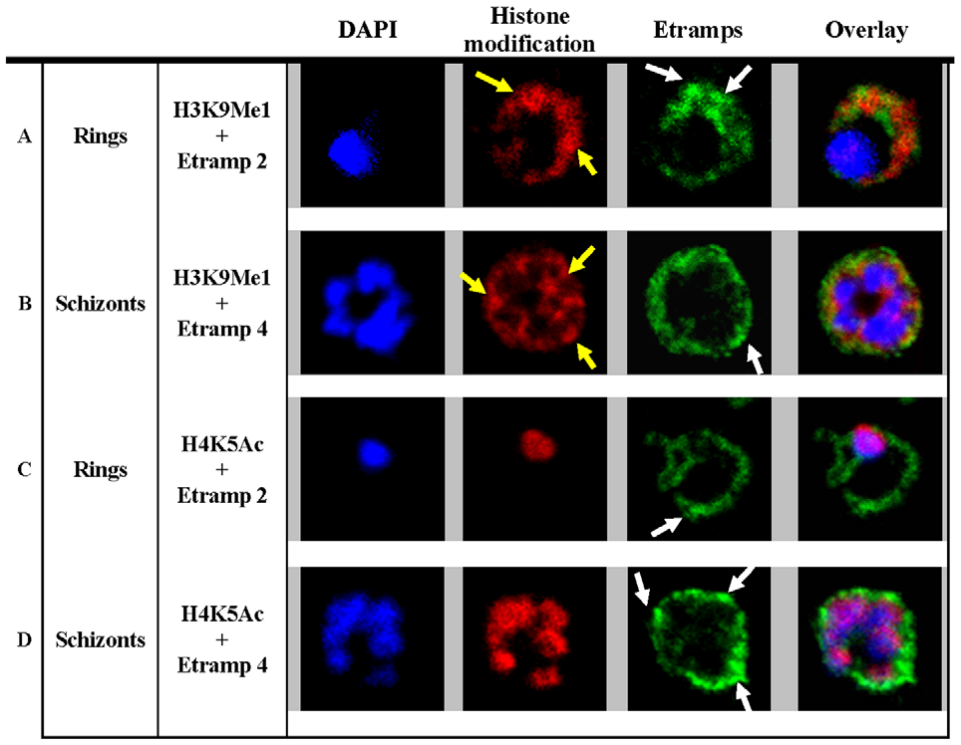
H3K9Me1 localized to the parasitophorous vacuole during the ring stage. Co-localization of H3K9Me1 with Etramp 2 (A) and 4 (B) was performed in ring and schizont stage parasites respectively. Similarly, co-localization of H4K5Ac with Etramp 2 (C) and 4 (D) was performed in ring and schizont stage parasites respectively. H3K9Me1/H4K5Ac and Etramp 2/4 were stained red and green respectively. DAPI stained nuclear DNA blue. Yellow and white arrows indicate foci of more intense fluorescence produced by H3K9Me1 and Etramp labeling respectively. In ring stage parasites, compared to schizonts, H3K9Me1 partially co-localized with Etramp 2 indicating localization to different compartments of the PV. H4K5Ac was localized solely to the nucleus and did not co-localize with either Etramp 2 or 4.

To explore this phenomenon further, we performed co-localization studies of H3K9Me1 with two well established parasitophorous vacuolar (PV) markers, Etramp 2 in the ring and Etramp 4 in the schizont stage [13]. The PV is a double membrane complex that consists of an inner parasite plasma membrane (PPM) and an outer second membrane termed parasitophorous vacuolar membrane (PVM). The Etramp proteins are integral proteins of the PVM organized in separate oligomeric arrays that delineate distinct microdomains in this membrane [14]. In agreement with this, both Etramp 2 and Etramp 4 exhibit a ring-like shape distribution that is consistent with their PVM localization (Figure 2). The non-uniform labeling of both Etramp proteins in the PV likely reflects the distinct microdomains present in this compartment (Figure 2, white arrows). In the ring stage, H3K9Me1 distribution showed a partial co-localization with Etramp 2, with most of the remaining signal outlining the same cellular region. The co-localization was only observed in the weaker staining areas while the strong signal foci observed for both H3K9Me1 (Figure 2A, yellow arrows) and Etramp 2 (Figure 2A, white arrows) were non-overlapping. This observation suggests that H3K9Me1 and Etramp 2 localized to different regions within the PV. A similar but somewhat less pronounced pattern of co-localization was observed between H3K9Me1 and the schizont PV marker, Etramp 4 (Figure2B). However, in this stage a substantial amount of H3K9Me1 signal was also observed in the parasite cytoplasm. For comparison, H4K5Ac showed a strictly nuclear distribution and did not co-localize with either of the PV markers at any developmental stage (Figure 2C and D).

### Biochemical studies of H3K9Me1 intracellular localization

**Figure 3:**
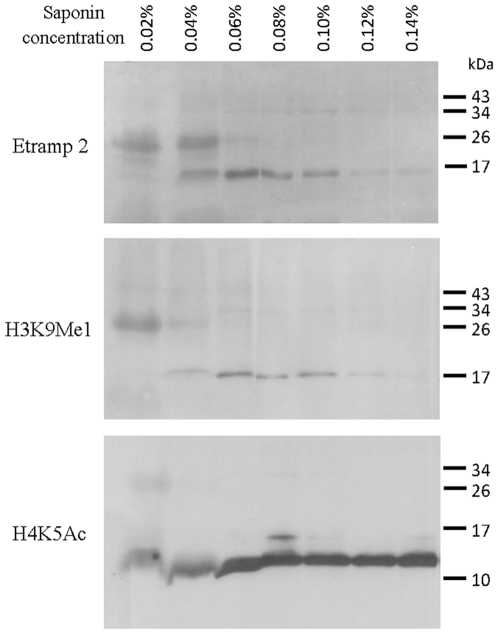
Immunodetection of Etramp 2, H3K9Me1 and H4K5Ac under different concentrations of saponin treatment. Ring stage *P. falciparum* infected red blood cells were subjected to different concentrations of saponin treatment, ranging from 0.06% to 0.14%. A strong signal corresponding to the expected molecular weights for Etramp 2 and H3K9Me1 was detected with 0.06%, 0.08% and 0.1% saponin. At higher saponin concentrations (0.12% and 0.14%), disruption of the PVM resulted in significant reductions in Etramp 2 and H3K9Me1 signal. In comparison, levels of H4K5Ac were unaffected by the saponin treatment. A faint band appeared at approximately 34 kDa due to non-specific reaction of the secondary antibody. Molecular weights are shown in kDa.

Similar to previous findings [11], immunodetection using the H3K9Me1 antibody resulted in an extremely weak signal in total protein lysates extracted from parasite cells isolated from their host cells by saponin lysis. It has been previously shown that besides the erythrocyte membrane, saponin lysis also results in the partial disruption of the PVM [15]. Therefore, we speculated that the vast majority H3K9Me1 could be lost from the PVM after saponin treatment. To explore this possibility, we subjected parasitized red blood cells to different concentrations of saponin, speculating that the milder saponin exposure will lead to lesser lysis of the PV. Indeed, milder lysis with 0.06%, 0.08%, and 0.10% of saponin allowed good detections of H3K9Me1 as well as the control markers: Etramp 2 (PV) and H4K5Ac (nuclei). This suggests that at these concentrations saponin can lyse the erythrocyte membrane but does not affect the PV dramatically. On the other hand, the western blot signal for both H3K9Me1 and Etramp 2 was significantly reduced in *Plasmodium* cells lysed with higher concentration of saponin (0.12%, and 0.14%) (Figure 3). In contrast, the signal for H4K5Ac that is localized solely in the nucleus, was unaffected by the different saponin concentrations. Taken together, H3K9Me1 and Etramp 2 antibody generated an identical pattern of the western blot signal across the set of total protein lysates generated from parasitized red blood cells with increasing concentrations of saponin. In agreement with our original hypothesis, this indicates that H3K9Me1 is localized in the PV.

**Figure 4:**
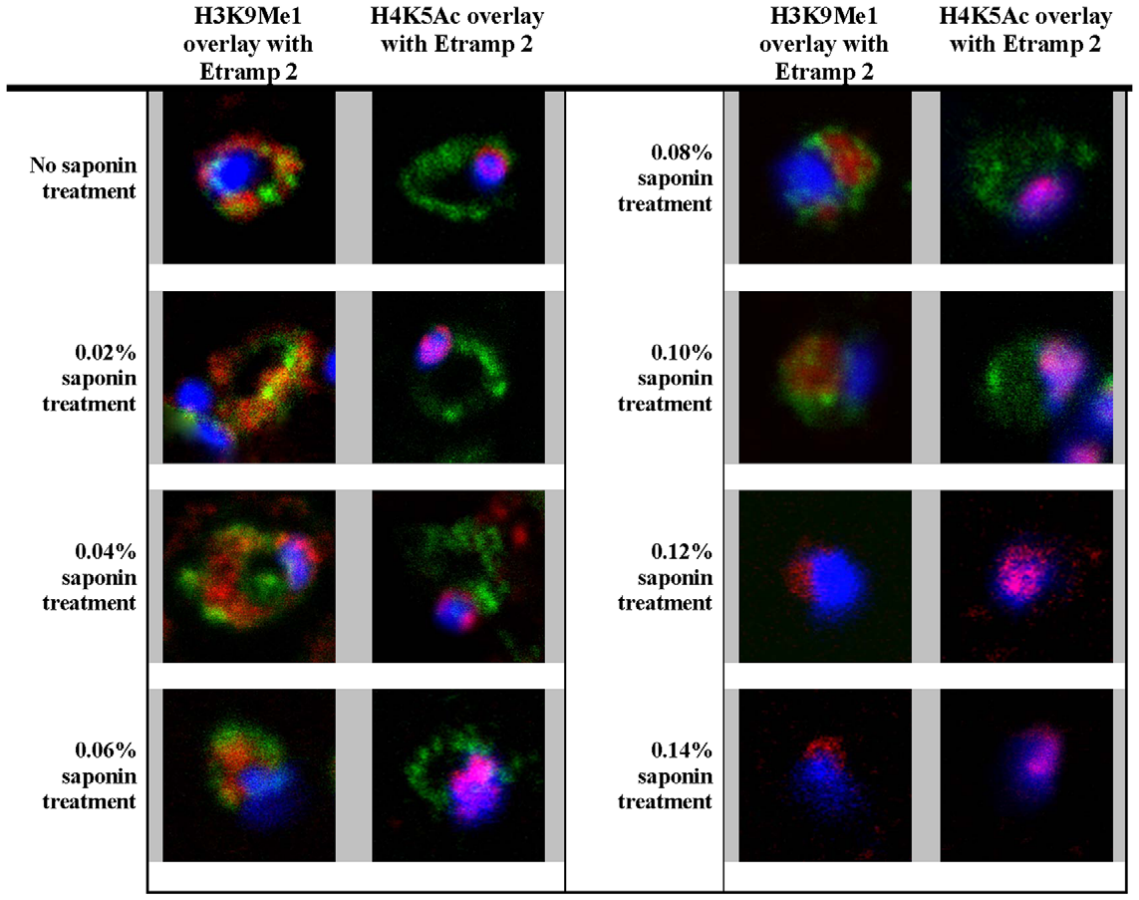
Loss of H3K9Me1 with disruption of the parasitophorous vacuole. *P. falciparum* infected red blood cells were subjected to various concentrations of saponin treatment and analyzed by IFA. H4K5 acetylation (red) remained co-localized with the DAPI stained nuclear DNA (blue) regardless of increasing concentrations of saponin. Etramp 2 (green) and H3K9Me1 (red) were gradually lost, resulting from the disruption of the PVM.

Importantly, the western blot signal detected by the H3K9Me1 corresponded to a protein with the predicted molecular weight of histone 3 (∼15.5 kDa), which indicates that the IF signal yielded by this antibody is not due to possible cross-reactivity to a different *P. falciparum* protein(s). To verify this further, we performed IF with the saponin lysed parasite samples used for the western blotting (Figure 4). At the lower concentrations of saponin (0.02% and 0.04%), the majority of the red blood cells were not lysed, and most of the parasitized red blood cells and the parasites remained intact. While the H3K9Me1 remained partially co-localized with Etramp 2, H4K5Ac antibody labeled the nuclear periphery as detected with our original IF experiments with intact cells. As the concentration of saponin increased (0.06% to 0.1%), the number of intact parasitized red blood cells and the number of intact parasites decreased. Although, the Etramp 2 and H3K9Me1 labeling in these parasites became weaker and more diffused, the partial co-localization is still evident. Interestingly, the increasing concentrations of saponin also affected morphology of the nuclei that appeared to be more diffused with H4K5Ac no longer concentrated at the nuclear periphery. These observations suggested that at this concentration saponin lysis disrupts the morphology of the whole parasite cell. At the highest concentration of 0.12% and 0.14% saponin, all red blood cells were lysed leaving only a small number of intact parasites. In these, we were unable to detect Etramp 2 which suggests that these concentrations of saponin cause a complete lysis of the PV with most of the proteins being washed away from the sample. Although a small amount of the H3K9Me1 signal could be detected just outside the nucleus, the vast majority of the H3K9Me1 labeling was also abolished (Figure 3).

### Localization of H3K9Me1 in other *Plasmodium* species

**Figure 5:**
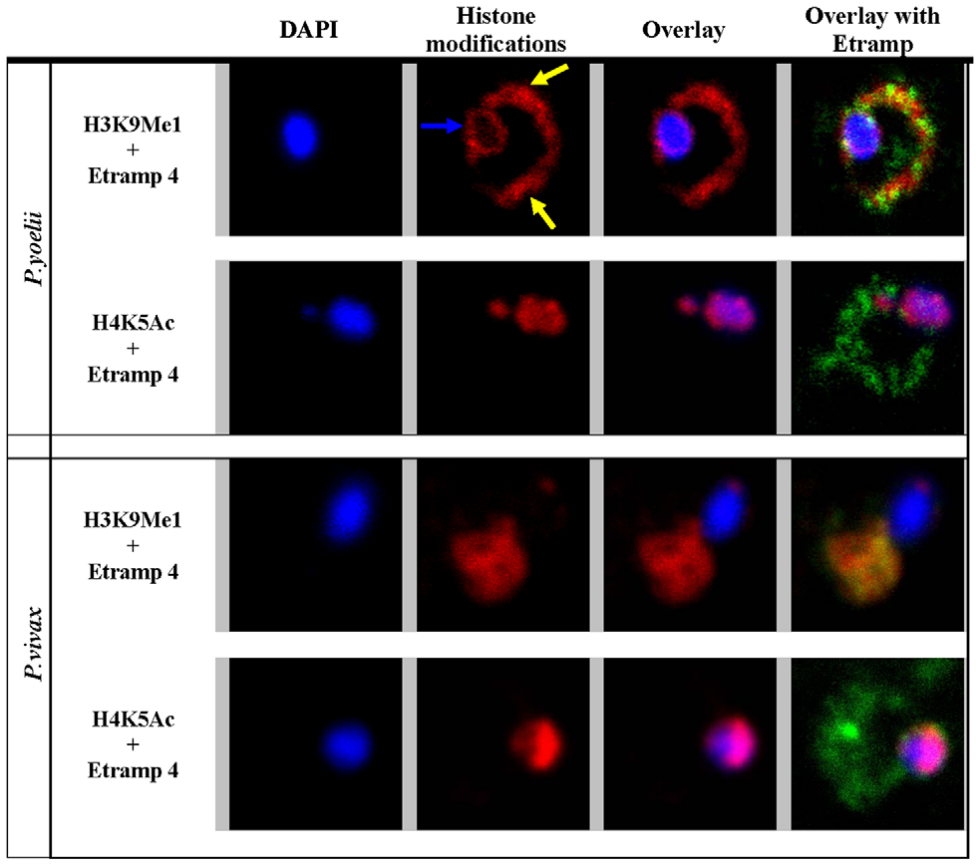
Immunofluorescence analysis of H3K9Me1 localization in *P. yoelii* and *P. vivax*. In both *P. yoelii and P. vivax* H3K9Me1 (red) co-localized with the PV marker Etramp 4 (green) whereas H4K5Ac (red) co-localized with the DAPI stained nuclear DNA (blue).

The localization of histone 3 outside the nucleus is highly surprising and it suggests that during evolution the *Plasmodium* histone 3 has acquired an additional function that is distinct from chromatin formation. To investigate this evolutionary event, we analyzed the H3K9Me1 localization in two related malaria parasite species: the rodent parasite *P. yoelii* and the second human malaria species *P. vivax* (Figure 5). In the *P. yoelii* ring stage parasites, obtained from *in vivo* growth in the mouse host, anti-H3K9Me1 staining produced a ring shaped pattern outside the nucleus that is similar to the signal observed in *P. falciparum* (Figure 1 and 2). IF labeling of the *P. yoelii* parasite with the *P. falciparum* Etramp 4 antibody exhibited a similar pattern that is reminiscent of the parasite periphery. Similar to *P. falciparum*, in *P. yoelii*, H3K9Me1 outlines a similar cellular region as Etramp 4 and in addition, partially co-localizes with this PVM marker. As opposed to the observations made in *P. falciparum*, anti-H3K9Me1 also labeled the nuclear periphery, producing a ring shape pattern which suggests a presence of this modification in the *P. yoelii* nuclei at higher levels (Figure 5, blue arrow). H4K5Ac labeled the nucleus and we did not observed any co-localization with Etramp 4. In the *P.vivax* ring/trophozoite stage parasites, anti-H3K9Me1 labeling was also detected outside of the nucleus and overlapped well with the IF signal from Etramp 4. Although the Etramp4 antibody were originally raised against the *P. falciparum* paralogue, the obtained IF signal on the parasite periphery indicates that this antibody recognizes at least one of the 10 and 9 Etramp orthologues in *P. yoelii* and P. vivax, respectively. Taken together these data suggest that the extranuclear localization of H3K9Me1 is conserved in *Plasmodium* species. This observation further strengthens the prediction that the PV localized histone 3 plays an important role in the *Plasmodium* parasite development in the erythrocytes.

### Co- localization of histone proteins with *P. falciparum* nucleoporin, Nup100

**Figure 6:**
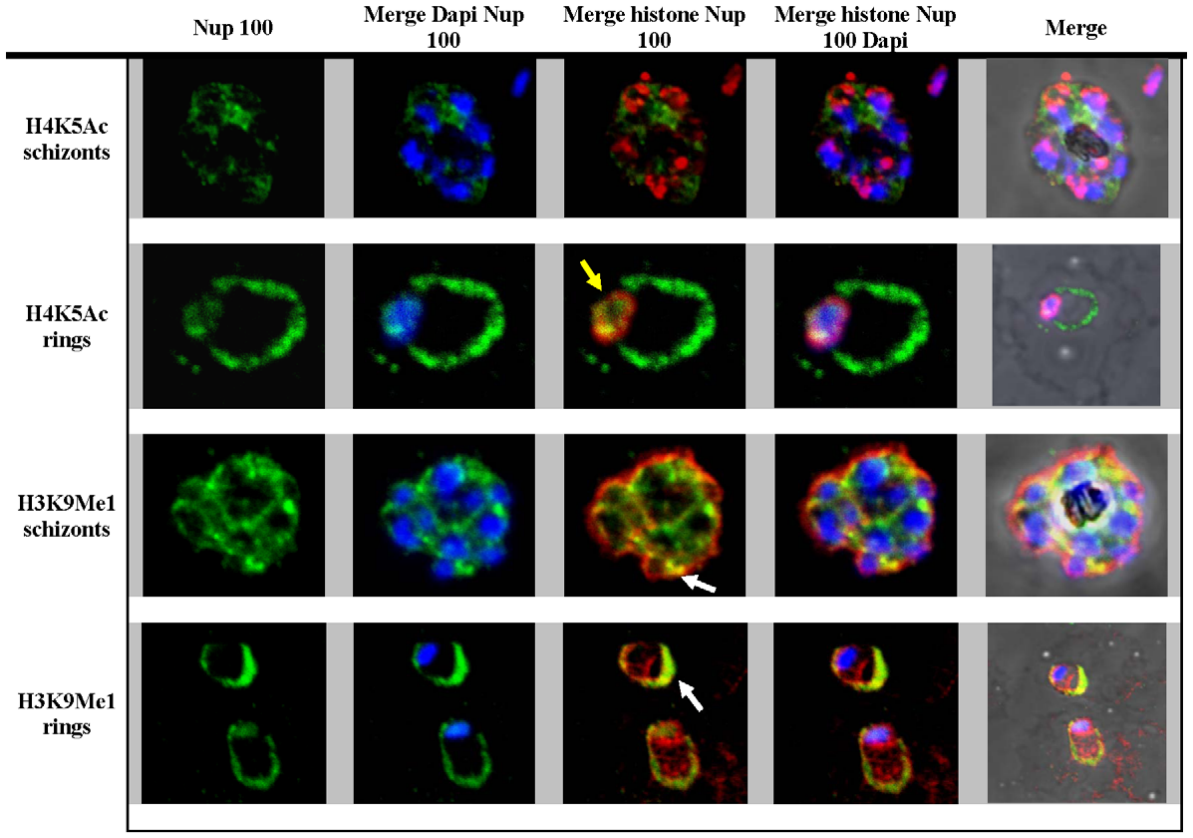
Immunofluorescence analysis of Nup 100. H3K9Me1, H4K5Ac and Nup 100 were stained red and green respectively. DAPI stained DNA blue. Yellow arrow indicates the co-localization of the H3K5Ac and Nup-100 in the ring stage parasites. White arrows indicate the co-localization of H3K9Me1 and Nup 100 outside the DAPI stained area, in the ring and schizont stage parasites.

Volz et al (2009) showed that many proteins associated with chromatin remodeling localize to distinct compartments at the nuclear periphery where they co-localize with the *P. falciparum* nucleoporin, Nup100 [16]. This result is consistent with the nuclear peripheral enrichment of the histone modifications associated with gene expression control (above). Using sequential IFA, we observe a considerable co-localization of H4K5Ac with Nup100 at the periphery of nuclei in the rings (Figure 6) and trophozoites (data not shown). However, a significant fraction of Nup100 appears to localize away from the nucleus. While in schizont Nup100 exhibits a discontinuous but disperse pattern in the cytoplasm, in rings, Nup100 distribution is reminiscent of PV localization. Remarkably, Nup100 exhibits a strong co-localization pattern with H3K9Me1 in both stages. In schizonts H3K9Me1 is more peripheral but there are areas of partial colocalization with Nup100 towards the cytoplasm (Figure 6). In the ring stage, these two proteins co-localize almost completely which indicates that part of Nup100 is also targeted to the PV. Taken together, these data suggest that nuclear periphery is potentially the main area of chromatin-dependent regulation of gene expression. However, it also shows that a significant portion of *Plasmodium* nuclear proteins is targeted away from the nuclei; in particular to the PV.

## Discussion

In this work, we characterized the intracellular distribution of 5 histone modifications that have not previously been investigated in *P. falciparum* (Figure 1). All four histone acetylations (H3K9Ac, H4K8Ac, H4K5Ac, and H4Ac4) exhibited a strong concentration around the nuclear periphery in a horseshoe-like pattern that has been previously demonstrated with H3K4Me3 [11]. With the exception of H4K8Ac, this pattern was conserved in all developmental stages of the IDC. In other eukaryotic species, these four acetylations are typically associated with epigenetic regulation of gene expression associated either with actively transcribed genes in euchromatin or silenced genes in heterochromatin (reviewed in [17]). The peripheral nuclear distribution of these histone modifications in *P. falciparum* is consistent with the emerging model of the spatial organization of epigenetic gene regulation in eukaryotic cells. According to this model, genes that are under strong epigenetic activation and/or suppression localize to distinct regions distributed along the nuclear periphery [18]. In *Saccharomyces cerevisiae*, many silenced genes are targeted to discrete domains along the nuclear envelope [19], while active genes are located in distinct chromatin domains that are anchored to the components of the nuclear pores [20]. Lopez-Rubio et al (2007) demonstrated the existence of at least one type of a gene silencing compartment at the periphery of the *P. falciparum* nuclei [4]. This discrete compartment is delineated by H3K9Me3 and is involved in silencing of the *var* gene family that is located at the chromosomal subtelomeres and encodes important factors for host-parasite interaction. The horseshoe-like pattern of immunofluorescence signal that was first demonstrated for H3K4Me3 [11] and reproduced for this and four other histone modifications in our work, may correspond to chromatin domains at the nuclear periphery that mediate epigenetic regulation of other (non-*var*) genes located in the intrachromosomal regions. There are several supporting evidences for this model. First, ChIP-chip analyses showed that both H3K4Me3 and H3K9Ac associate with a large number of intrachromosomal loci distribution across the *P. falciparum* genome but is absent from the heterochromatin loci at the chromosome ends [4]. Second, H3K9Ac has been shown to be associated with actively transcribed genes in the schizont stage of the *P. falciparum* IDC [6]. Third, in our recent work, we show that inhibitors of histone deacetylases cause dramatic changes in the chromosomal distribution of H4K8Ac, H4Ac4, H3K9Ac and H3K4Me3 that is linked with transcriptional deregulation of genes in these loci [21]. Together with the abovementioned results, our data suggest that the nuclear periphery is one of the most active areas in epigenetic regulation of gene expression. Future studies will be needed to fully understand the role of this sub-cellular compartment in the transcriptional regulation in the human malaria parasites.

The most intriguing finding of this work is the localization of H3K9Me1 to the parasite periphery that was confirmed by co-localizations as well as differential saponin treatments. This localization is surprising as histones are considered to be amongst the most conserved proteins in eukaryotic cells and their role is linked with the chromatin structure. Nonetheless, extra-nuclear and extra-cellular localization of histones have been reported in other species. In humans, histone H1 was detected in the cytoplasm of the small intestinal epithelial cells and upon their programmed cell death, H1 was found also in the extracellular environment [22]. The intra and extracellular H1 was found to possess antimicrobial activity preventing microbial penetrations into the villus epithetial cells [22]. In stimulated T cells, two histone proteins H2B and H3 were found on the surface binding specifically to dextrin-2-sulphate, a sulphated polysaccharide that inhibits invasion of the human immunodeficiency virus type 1 [23]. H1.2, an isoform of the linker histone H1, was also shown to translocate from the nucleus into the cytoplasm and induced the release of proapoptotic factor, cytochrome C, from the mitochondria to bring about cell death [24]. Besides the linker H1, the nucleosome core histones were also found to be released from the nuclei at early stages of apoptosis [25]. Even in the nuclei, histones have adopted other non-canonical functions. The histone variant H2AX plays functional role in DNA repair and genome stability and hence cancer susceptibility [26] while histone H2B was reported to be involved in the post-replication DNA repair [27]. Overall, these observations indicate that despite their strict evolutionary conservation, histones can acquire other biological functions that are distinct from formation of the nucleosome particle. The localization of H3 to the *P. falciparum* PV likely represents another case of such functional diversification.

The extra-nuclear localization of Nup100 in the schizont stage likely reflects the biogenesis on the nuclear envelop (NE) during the mitotic division. It was previously shown that during eukaryotic cell division the membranes of NE are ruptured and re-absorbed into the surrounding endoplasmic reticulum (ER) [28]. During this time, the ER serves as a mitotic storage site for NE proteins and subsequently as a precursor for formation of new NE for newly formed daughter cells [29]. The dispersed localization pattern of Nup100 during the schizont stage likely represents this process when *Plasmodium* NE proteins are retracted into the ER during the multiple rounds of DNA replication in the schizont stage. The ER is likely an assembly site of the newly synthesized components of NE that has to be produced in sufficient quantities for up to 32 new daughter cells generated during plasmodium schiozogony. In our study, we find that after the replication and subsequent invasion, a proportion of Nup100 is transported to the PV. Here it is co-localized with H3K9Me1 another protein whose main function is associated with the nuclei. Although it is unclear how are these proteins transported to the PV, their partial co-localization in the schizont stage may reflect a common transport pathway that may involved a fraction of ER derived vesicles. It will be interesting to understand whether this transport represents a generic “overflow” of highly abundant precursors of nuclear formation or a specific transport mechanism that enables some of the nuclear proteins to fulfill a specific function, distinct from their original role in the nuclei.

The PV is created during merozoite invasion. It provides an enclosed environment for parasite development and facilitates nutrient and protein transport between the parasite and host cytoplasm [30]. However, the molecular basis of PV formation, maintenance, and many other aspects of its function are poorly understood. Although, a number of proteins including the Etramp family have been localized to the PV, their molecular functions remain largely uncharacterized [13], [14]. Like these proteins, the functional role of H3 in the PV remains to be investigated, however, this localization indicates its “moonlighting function”. Moreover, this function is in some way linked with mono-methylation of H3 at the lysine residue 9. Alternative (moonlighting) functions were previously reported for at least two additional highly abundant *Plasmodium* proteins. First, *P. falciparum* aldolase (PfAld) has been implicated in forming a physical bridge between the surface adhesins and the actin cytoskeleton during the invasion process [31]. This interaction is particularly peculiar since PfAld belongs to the class I aldolases that are highly conserved and in the vast majority of eukaryotic organisms involved strictly in glycolysis. Interestingly, the aldolase involvement in invasion appears to be evolutionarily conserved in most (if not all) *Plasmodium* species [31] as well as the related apicomplexan *Toxoplasma gondii* [32]. Second example is enolase (Pfen), that similarly to aldolase, is a highly conserved glycolytic enzyme but in *P. falciparum* it also localizes in the nucleus, food vacuole, and cytoskeleton and plasma membrane (including the surface of the invasive merozoites) [33]. Antibodies against this protein are able to protect mice against infection with *P. yoelii* which suggest the role of Pfen in host parasite interactions [34]. A recent proteomic study showed that Pfen undergoes extensive posttranslational modifications [35]. This suggests that specific modifications may mediate the different Pfen localizations and may be also essential for the putative roles of Pfen in these compartments. Similar to PfAld and Pfen, H3 is a highly conserved protein that is expressed at high levels during the schizont stage when most of the specialized molecular mechanisms associated with invasion of the new host erythrocytes are being formed. It is tempting to speculate that due to their high abundance and thus ubiquitous presence, these proteins evolved new functions in the highly specialized molecular processes unique to the *Plasmodium* parasites.

## Materials and Methods

### Parasite culture

*P. falciparum* 3D7 strain was cultured as described [36]. Briefly, parasites were cultured in human red blood cells in RPMI 1640 media supplemented with 0.25% Albumax (Invitrogen), 2 g/l sodium bicarbonate, 50 µl/l gentamycin, and 0.1 mM hypoxanthine. The culture was maintained at 2% hematocrit. Parasites were synchronized by 2 consecutive 5% sorbitol treatments 8 h apart for 3 generations before analysis. The *P. falciparum* parasites were cultured in human erythrocytes purified from whole blood obtained from anonymous donors who signed a written consent. The blood donation scheme for these studies was approved by the internal ethics committee of the Nanyang Technological University. Details of the P. vivax sample used for the blood smears were previously reported [37]. Collection of the P. vivax samples was approved by the ethics committee of Mahidol University, Bangkok, Thailand.

### Lysis of red blood cells and harvest of parasites

Parasitized red blood cells at 5% parasitemia were harvested for analysis. Different concentrations of saponin (0.02% to 0.14%) were added to the red blood cells in a 10∶1 ratio, and allowed to lyse the red blood cells for 5 minutes at room temperature. The parasites were pelleted by centrifugation and washed twice with phosphate buffered saline (PBS).

### Western analysis

Parasite pellets were lysed by boiling in Laemmli sample buffer. Protein concentration was quantified by BioRad Protein assay (BioRad). Total protein lysate were separated on a 15% SDS PAGE and transferred onto nitrocellulose membrane. Primary antibodies against histone modifications were from Upsate and used at the following dilutions: H3K9Me1 1∶3000, H4K5Ac 1∶5000. The Etramps antibodies were a kind gift from Dr Tobias Spielmann (Bernhard Nocht Institute for Tropical Medicine, Germany) and used at 1∶1000 dilution. Horseradish peroxidase conjugated secondary antibodies (GE Healthcare) were used at 1∶4000.

### Immunofluorescence

Cultures were washed twice in PBS and smears of the cultures were prepared on microscopic slides. Immunofluorescence was performed as described [38]. Briefly, the cells were fixed in 4% paraformaldehyde/0.0075% glutaraldehyde for 30 min, quenched with 1 mg/ml NaBH4/PBS for three times for 15 min, and blocked with 5% BSA. Primary antibodies incubation was carried out overnight at 4°C at the following dilutions: H3K9Me1 1∶600, H4K5Ac 1∶1000, Etramp 2 1∶200, Etramp 4 1∶100, Nup100 1∶200. Fluorophore conjugated secondary antibodies from Invitrogen were used at the following dilutions: AlexaFluor 594 goat anti-rabbit 1∶1000, AlexaFluor 488 goat anti-rabbit 1∶1000 AlexaFluor 488 goat anti-rat 1∶500. Parasite nuclei were stained with DAPI. The slides were analyzed by Carl Zeiss LSM 510 Confocal Laser Scanning Microscope.
